# Exposure to Advertisement Calls of Reproductive Competitors Activates Vocal-Acoustic and Catecholaminergic Neurons in the Plainfin Midshipman Fish, *Porichthys notatus*

**DOI:** 10.1371/journal.pone.0070474

**Published:** 2013-08-06

**Authors:** Christopher L. Petersen, Miky Timothy, D. Spencer Kim, Ashwin A. Bhandiwad, Robert A. Mohr, Joseph A. Sisneros, Paul M. Forlano

**Affiliations:** 1 Department of Biology, Brooklyn College, City University of New York, Brooklyn, New York, United States of America; 2 Department of Psychology, University of Washington, Seattle, Washington, United States of America; 3 Virginia Bloedel Hearing Research Center, Seattle, Washington, United States of America; 4 Aquatic Research and Environmental Assessment Center, Brooklyn College, Brooklyn, New York, United States of America; 5 Programs in Neuroscience, and Ecology, Evolution, and Behavior, The Graduate Center, City University of New York, New York, New York, United States of America; Claremont Colleges, United States of America

## Abstract

While the neural circuitry and physiology of the auditory system is well studied among vertebrates, far less is known about how the auditory system interacts with other neural substrates to mediate behavioral responses to social acoustic signals. One species that has been the subject of intensive neuroethological investigation with regard to the production and perception of social acoustic signals is the plainfin midshipman fish, *Porichthys notatus*, in part because acoustic communication is essential to their reproductive behavior. Nesting male midshipman vocally court females by producing a long duration advertisement call. Females localize males by their advertisement call, spawn and deposit all their eggs in their mate’s nest. As multiple courting males establish nests in close proximity to one another, the perception of another male’s call may modulate individual calling behavior in competition for females. We tested the hypothesis that nesting males exposed to advertisement calls of other males would show elevated neural activity in auditory and vocal-acoustic brain centers as well as differential activation of catecholaminergic neurons compared to males exposed only to ambient noise. Experimental brains were then double labeled by immunofluorescence (-ir) for tyrosine hydroxylase (TH), an enzyme necessary for catecholamine synthesis, and cFos, an immediate-early gene product used as a marker for neural activation. Males exposed to other advertisement calls showed a significantly greater percentage of TH-ir cells colocalized with cFos-ir in the noradrenergic locus coeruleus and the dopaminergic periventricular posterior tuberculum, as well as increased numbers of cFos-ir neurons in several levels of the auditory and vocal-acoustic pathway. Increased activation of catecholaminergic neurons may serve to coordinate appropriate behavioral responses to male competitors. Additionally, these results implicate a role for specific catecholaminergic neuronal groups in auditory-driven social behavior in fishes, consistent with a conserved function in social acoustic behavior across vertebrates.

## Introduction

Vocalizations are key components of social behavior in many vertebrates. Perception of these social acoustic signals may elicit communicative responses and/or reproductive behavior in conspecifics and is essential for mating in many teleost fishes [1], [2], anuran frogs [3], and songbirds [4]. Various forms of vertebrate sociality including but not limited to consummatory and appetitive sexual behavior and aggression are mediated by a highly conserved and reciprocally connected suite of nuclei within the basal forebrain and midbrain termed the social behavior network (SBN) [5]–[7]. Catecholaminergic circuitry, including the ascending dopaminergic system, has been suggested to work in concert with the SBN to assess the salience of socially relevant stimuli, and to reinforce appropriate behavioral responses to such social stimuli [7], [8]. Interestingly but not surprisingly, key components of the neural circuitry that underlie vocal-acoustic behavior reside in the SBN and are highly conserved throughout vertebrate taxa [6], [9]. Although the neural circuitry responsible for encoding acoustic stimuli is well delineated in several vertebrate taxa, very little is known about how the auditory system interacts with the SBN to mediate responses to social acoustic signals. The neural substrates that allow vertebrates to produce vocalizations share similar developmental origins and vocal-acoustic communication is thought to have first evolved in teleost fishes, the most speciose vertebrate group [10]. As such, elucidating the interaction between the SBN and auditory circuitry in teleosts will provide a better understanding of the function and evolution of these systems in other vertebrates.

One teleost system that has become a powerful neuroethological model for investigating vocal-acoustic behavior among vertebrates is the plainfin midshipman fish (*Porichthys notatus*), in part because the production and perception of social acoustic signals is essential to the reproductive success of this species [1], [11]–[13]. The plainfin midshipman is a marine teleost fish in the family Batrachoididae that migrates seasonally (spring/summer) from deep offshore sites to spawn in rocky intertidal zones off of the northwest coast of the United States [11]. Type I or “nesting” males produce a long duration (>1 min) vocalization or “hum” that serves as an advertisement call to attract females. Females find courting males by localizing the hum, spawn once by depositing eggs on the roof of the type I male’s nest, and then return to deep offshore sites [14]. After fertilization, type I males guard their nest and continue to vocally court females for additional spawnings throughout the breeding season. As multiple type I males establish nests in close proximity to one another, an acoustic environment with concurrent overlapping hums is created whereby resident nesting males may be able to access and respond acoustically to the hums of neighbors [1]. The perception of potential competitors may affect an individual’s motivational state and subsequently elicit appropriate social responses.

In teleost fishes, the encoding of auditory stimuli first occurs in the saccule [15]–[17], the main endorgan of hearing in midshipman and most other teleosts, which projects via the eighth nerve to the descending octaval (DO) nucleus in the auditory hindbrain [18]–[20]. The rostral intermediate division of DO (DOri) is not only a major source of ascending afferent projections to the midbrain torus semicircularis (TS), but also projects to the prepacemaker nucleus of the vocal pattern generator [9], [18], [19] and thus provides a vocal-acoustic interface at the level of the auditory hindbrain in the medulla. Nucleus centralis within TS (TSnc) is largely medial and periventricular, and has been physiologically identified as an auditory center which can encode concurrent hums [21], [22] and shares reciprocal connections with the central posterior nucleus (CP, auditory thalamus) [9], [19] as well as with the anterior tuberal hypothalamus (AT). Finally, CP has reciprocal connections with AT and the ventral tuberal hypothalamus (vT), both of which are part of the descending vocal-motor circuitry and SBN [6], [9].

In the closely related Gulf toadfish, *Opsanus beta* (same family as the midshipman fish), exposure to conspecific advertisement calls is known to increase vocal production and raise circulating blood levels of 11-ketotestosterone and cortisol [23]. While acoustic playback challenges are known to elicit simultaneous changes in circulating hormone levels, vocal behavior and territoriality [24], the physiological response to such a challenge may not be limited to changes in steroid levels alone. Catecholamines (CA), which include dopamine (DA) and noradrenaline (NA), are highly conserved neurochemicals that modulate motivated behavior and sensory perception across vertebrates [25], [26]. The locus coeruleus (LC) is NAergic, found in the isthmal brainstem of all vertebrates [27] and is an important regulator of behavioral arousal and sensory systems, including audition [28]–[30]. In teleosts, DAergic neurons in the periventricular posterior tuberculum (TPp) appear to be homologous to the amniote A11 CA group which sends descending projections from the diencephalon to the spinal cord [31], [32]. Neurons within TPp also display ascending projections to the ventral telencephalon which originally lead to the proposal of similarities to the A10 ventral tegmental neurons of the tetrapod mesolimbic system [8], [33]. Interestingly, both A10 and A11 DAergic neurons are known to be important in motivated social and sexual behavior in other species [34]–[37]. However, the function of these CA groups and whether or not they are important modulators of social behavior in teleost fishes is unknown. In midshipman, like other teleosts, connectivity of DAergic TPp neurons and TPp in general [38] appear to make them ideal candidates for sensorimotor (including auditory-vocal) integration and higher order decision making [32], [39].

The goal of this study was to characterize how the brain of a vocal fish responds during exposure to the advertisement calls of potential competitors. To this end we presented wild-caught type I midshipman males (*P. notatus*) with playbacks of advertisement calls of field-recorded midshipman, and examined patterns of neural activity by assaying changes in cFos, an immediate early gene product, within the CNS. Specifically, we tested the hypothesis that type I male midshipman exposed to other advertisement calls would show elevated neural activity (i.e., increased cFos response) in auditory and vocal-acoustic brain centers as well as differential activation of CA neurons compared to control males exposed only to ambient noise. We quantified cFos immunoreactive (-ir) neurons in major auditory nuclei including DOri, TSnc, and CP, as well as in two vocal-acoustic centers (AT, vT) which are both part of the teleost SBN. We also examined whether the social acoustic signals increased the cFos response in CA neuronal populations in the LC and TPp by double labeling with tyrosine hydroxylase (TH), the rate-limiting enzyme in CA synthesis. Our results are the first to demonstrate a link between the exposure to social acoustic signals and the activation of specific catecholaminergic nuclei along with brain regions associated with auditory processing and social behavior in a teleost fish. In addition, our data support the hypothesis that CAs are important neurochemicals involved in the modulation of auditory-driven social behavior across vertebrates.

## Materials and Methods

### Ethics Statement

All experimental animal procedures performed in this study were approved by the Institute for Animal Care and Use Committee of the University of California, Davis (Protocol Number: 15977), and animals were collected from the field under California Department of Fish and Game Permit 802021-01.

### Animals

Male plainfin midshipman fish (*Porichthys notatus*) were collected from nests during the morning low tides at several natural breeding locations in Tomales Bay near Marshall, CA, USA, in the same geographical locations used in previous studies over the last 20 years (e.g., [40]–[42]). Type I males were distinguished from type II’s [11] and females by size, and sex was confirmed post sacrifice. Type I males were transferred to coolers with aerated sea water and then transported to the UC Bodega Marine Laboratory (BML) in Bodega Bay, CA where they were housed in flow-through sea water aquaria until play-back trials within 24–72 hrs of collection. Holding time between collection and playback should not have affected the fishes’ sensory capabilities as previous studies showed no decrease in female midshipman auditory sensitivity to encoding frequencies of the male mate call until more than 25 days post-collection [16]. At BML, the fish were maintained in large communal tanks at natural ambient temperatures (12–14°C) until playback experiments were conducted.

### Playback Experiment

All tests were conducted at BML in an outdoor, cylindrical concrete tank (4 m diameter, 0.75 m depth) at night between 9 pm and 1∶00 am during July 2011. A monopole sound source (Lubell AQ339, Clark Synthesis, Littleton, CO, USA) was suspended from a wooden beam in the center of the tank and positioned 10 cm above the tank floor. Animals were placed in a 30 cm diameter plastic mesh cylinder cage in the tank at a fixed distance from the underwater sound source so that the average peak sound level would be 130 dB (re 1 µPa) at the center of the cage when the sound stimulus was turned on. The calibrated stimulus sound levels of 130 dB (re 1 µPa) are consistent with the sound pressure levels of the advertisement calls produced by type I males recorded near their nests [43]. Sound levels were calibrated and measured nightly using a mini-hydrophone (model 8103, Brüel and Kjaer, Norcross, GA, USA), a charge amplifier (Brüel & Kjaer model 2692) and oscilloscope. Sound playback males (n = 6) were subjected to a 30 minute playback of 5 looped field-recorded advertisement calls of other male midshipman. Control males (n = 6) were placed in the same arena for 30 minutes at the same time of night with only ambient noise (∼100 db re 1 µPa). Animals were sacrificed 120 minutes post trial by deep anesthetization in 0.025% benzocaine in seawater followed by transcardial perfusion with ice cold teleost ringers followed by 4% paraformaldehyde in 0.1 M phosphate buffer (PB; pH 7.2). We chose 120 minutes to sacrifice because Okuyama et al. [44] demonstrated that levels of cFos protein were significantly elevated in the medaka fish (*Oryzius latipes)* brain starting at 60 minutes after being treated with pentylenetetrazol (PTZ) a GABA antagonist, and remain elevated for up to 150 minutes. Prior to sacrifice, standard length was measured from the tip of the snout to the caudal peduncle. We calculated gonadosomatic index as the percentage of gonad to body mass. Brains were harvested, post-fixed for 1 hour, and stored at 4°C in 0.1 M PB in 0.03% sodium azide until sectioned. Approximately 24 hours prior to sectioning, brains were cryoprotected in 30% sucrose-PB solution. Brains were sectioned on a cryostat in the transverse plane at 25 µm in 2 series and collected onto subbed slides and stored at −20°C until labeling. For this experiment, 1 of 2 series was used for immunohistochemistry.

### Immunohistochemistry

Slides were allowed to warm to room temperature and were washed 3 times for 15 minutes in phosphate buffered saline (PBS; pH 7.2) followed by 1 hour soak in 0.3% Triton X-100 in PBS (PBST) +10% blocking solution [(8% normal donkey serum (DS, Jackson Immunolab, West Grove, PA)+2% bovine serum albumin (Sigma-Aldrich, St. Louis, MO) (PBST-DS/BSA)]. After blocking, tissue was incubated for 16 hours at room temperature in PBST-DS/BSA containing mouse anti-tyrosine hydroxylase (TH, 1∶1000 Millipore, Temecula, CA) and rabbit anti-cFos (1∶2000 Santa Cruz Biotechnology, Santa Cruz, CA). Post incubation, slides were washed 5 X 10 min in PBS +0.5% normal donkey serum (PBS-DS). This was followed by 2 hour incubation in PBST-DS/BSA+donkey anti-mouse conjugated to Alexa Fluor 488 (dilution 1∶400, Life Technologies, Norwalk, CT), and donkey anti-rabbit conjugated to Alexa Fluor 568 (1∶200, Life Technologies). Slides were then washed 4 X 10 min in PBS, and coverslipped with ProLong Gold with DAPI (Life Technologies), and allowed to dry in the dark at room temperature for approximately 48 hours at which time they were sealed with nail polish and stored at 4°C. Specificity for both the TH [45], [46] and cFos [44], [47] antibodies have been demonstrated elsewhere in teleost fishes. Additionally, controls without primary or secondary antibodies or preabsorption of cFos primary antibody with its blocking peptide (SC-253P, Santa Cruz Biotechnology) as per manufacturer’s instruction, eliminated specific fluorescent signal.

### Image Acquisition and Anatomy

#### Auditory/Vocal-Acoustic centers

Images were acquired on an Olympus BX61 epifluorescence compound microscope (Tokyo, Japan) using MetaMorph imaging and processing software (Molecular Devices, Sunnyvale, CA). Each nucleus analyzed in the auditory/vocal-acoustic system was identified at low magnification and photomicrographed with a 20x objective at the same light level and exposure time. Each photomicrograph was taken consecutively using Texas Red and DAPI filter sets (Chroma, Bellow Falls, VT) within a z-stack at 5 levels (2 levels above and 2 levels below a central plane), each 1 µM thickness. These 5 photomicrographs were combined into a single projected image in MetaMorph. For each image, cFos-ir signal was thresholded above background [48], and the number of cFos-ir cells was quantified using MetaMorph’s integrated morphometry analysis (IMA) feature. A size filter in IMA was employed to count only immunofluorescence ≥125 pixels. This value was determined *a priori* as the average pixel count for the smallest size of cFos-ir cells we considered to be signal. cFos-ir cells that were clumped were counted manually: a region was created around the cFos-ir cell(s) in question, and transferred to the DAPI channel where the presence of multiple nuclei was confirmed. Sampling strategy was determined per region to account for intrinsic variation in size between nuclei [49]. In the case of tissue loss or damage, the opposite side of the brain was used (for unilateral sampling), or the section was omitted (see below). In each animal, the average number of cFos-ir neurons per section was calculated per nucleus. Experimenter was blind to treatment conditions of all slides analyzed.

**Figure 1:**
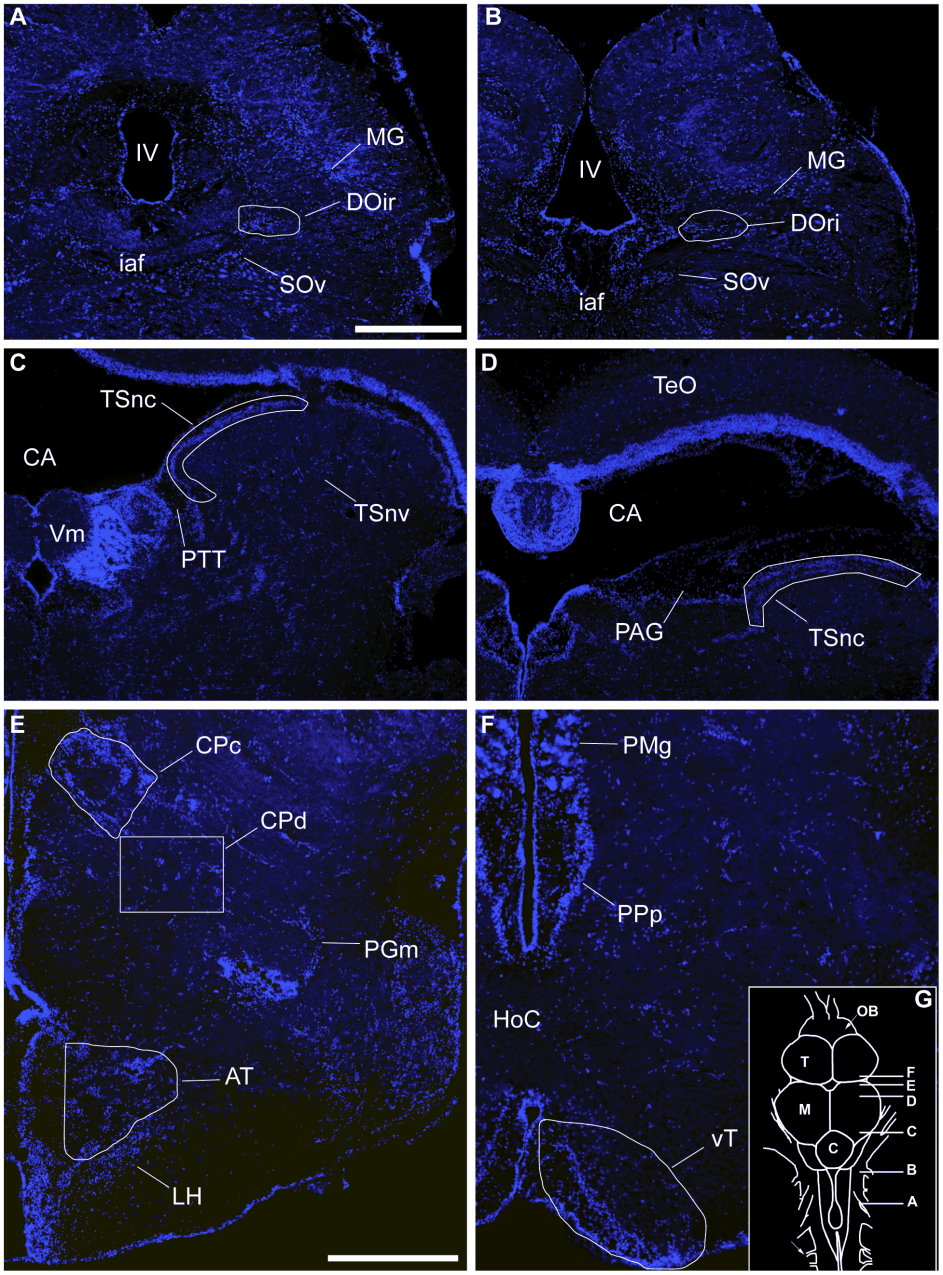
Auditory and vocal-acoustic anatomy. Transverse sections with DAPI nuclear counterstain (blue) showing white borders around nuclei in which numbers of cFos-ir neurons were quantified within auditory and vocal-acoustic pathways. The caudal (**A**) and rostral (**B**) extents of the rostral intermediate descending octaval nucleus (DOri). The caudal (**C**) and rostral (**D**) extents of the periventricular nucleus centralis of the torus semicirularis (TSnc). (**E**) Compact (CPc) and diffuse (CPd) divisions of the central posterior nucleus. Ventromedial to CP is the anterior tuberal nucleus (AT) in the ventral hypothalamus. (**F**) Ventral tuberal nucleus (vT) of the anterior hypothalamus. (**G**) (inset) Dorsal view of midshipman brain with relative positions of A–F. **Abbreviations:** Cerebellum (C); Cerebral Aqueduct (CA); Horizontal commissure (HoC); Internal arcuate fiber tract (iaf); Fourth ventricle (IV); Lateral hypothalamus (LH); Midbrain (M); Magnocelluar octaval nucleus (MG); Olfactory bulb (OB); Medial nucleus preglomerulosus (PGm); Periaqueductal gray (PAG); Magnocellular preoptic nucleus (PMg); Posterior parvocellular preoptic nucleus (PPp); Paratoral tegmentum (PTT); Optic tectum (TeO); Ventral secondary octaval nucleus (SOv); Telencephalon (T); Molecular layer of the valvula (Vm). Scale Bars = 500 µm.

The most basal level of the ascending auditory pathway we analyzed was the rostral intermediate division of the descending octaval nucleus (DOri). Beginning rostrally in DOri, (Figure 1A) we sampled serial sections caudally until its disappearance at the level of the octavolateralis efferent nucleus (OE) (Fig. 1B) [18], [19]. A border was drawn around DOri in the DAPI channel and transferred to the Texas red channel to quantify the number of cFos-ir neurons. Landmarks from published descriptions of this nucleus were used to identify its extent [19]. As DOri is directly innervated by nVIII [19], we quantified cFos-ir neurons bilaterally to account for any activation bias that may have occurred due to the position of the fish relative to the speaker. On average, 10.8 sections were analyzed (±3.7 SD). An independent samples t test was performed to determine that there was no difference in number of sections used between groups (*p*>0.83).

In the auditory midbrain, we quantified numbers of cFos-ir neurons within an area drawn around the periventricular TSnc (Figure 1C–D) in 2 adjacent photomicrographs captured with 20x objective at each level sampled. Medially, this area excluded the periaqueductal gray (PAG) and paratoral tegmentum (PTT) which are both known to elicit vocal behaviors in physiological preparations [9]. Laterally, landmarks were used to assure that there was no overlap in photomicrographs. We began analysis of TSnc at the level of the molecular cell layer of the valvula (Vm). Moving in the caudal to rostral direction, we sampled every fourth section unilaterally until the appearance of the auditory thalamus (CP). The right side of the brain was used unless there was damage to the tissue, in which case we used the left side. Five sections throughout TS were used to calculate average cFos-ir neurons per section, except for 1 animal in the social signal group where 4 sections were used. Toral efferents project to both the compact (CPc) and diffuse (CPd) divisions of the central posterior nucleus (CP) of the thalamus (Figure 1E) [19]. CPc forms a wing-shaped band of cells lateral to the midline, and CPd is a loose collection of cells which extend ventrolaterally from CPc [9]. A boundary was drawn around CPc, and cFos-ir neurons were quantified within. As CPd is by nature diffuse we quantified cFos-ir neurons in the entire 20x image, starting at the ventrolateral extent of CPc. We sampled CP unilaterally on the right side of the brain in 3 consecutive sections. One photomicrograph was taken for each division, and we analyzed cFos-ir neurons within CPc and CPd for each section analyzed. Additionally, we summed cFos-ir neurons within CPc and CPd for a comparison of total activity within the auditory thalamus. There were no differences in number of sections analyzed between groups in either TS or CP (independent samples t test, *p*>0.35 in both cases).

AT is located in the ventral hypothalamus, rostral to the dorsal periventricular hypothalamus (Hd) and dorsal to the lateral hypothalamus (LH; Figure 1E) [50]. We drew a boundary around AT in the DAPI channel which was then transferred to the Texas red channel where numbers of cFos-ir neurons were quantified. In AT, we sampled 3 consecutive sections unilaterally on the right side of the brain. There was no difference in number of sections analyzed between groups (independent samples t test, *p* = 0.18). vT was sampled unilaterally in serial sections on the right side of the brain starting at the level of the horizontal commissure (Figure 1F) [9], [50]. An area was drawn around vT in the DAPI channel and transferred to the Texas red channel where the number of cFos-ir neurons was quantified within. On average, 2.7 (±1 SD) sections were used per animal, and an independent samples t test showed that there was no difference in number of sections analyzed between groups (*p*>0.15).

#### Catecholamines

**Figure 2:**
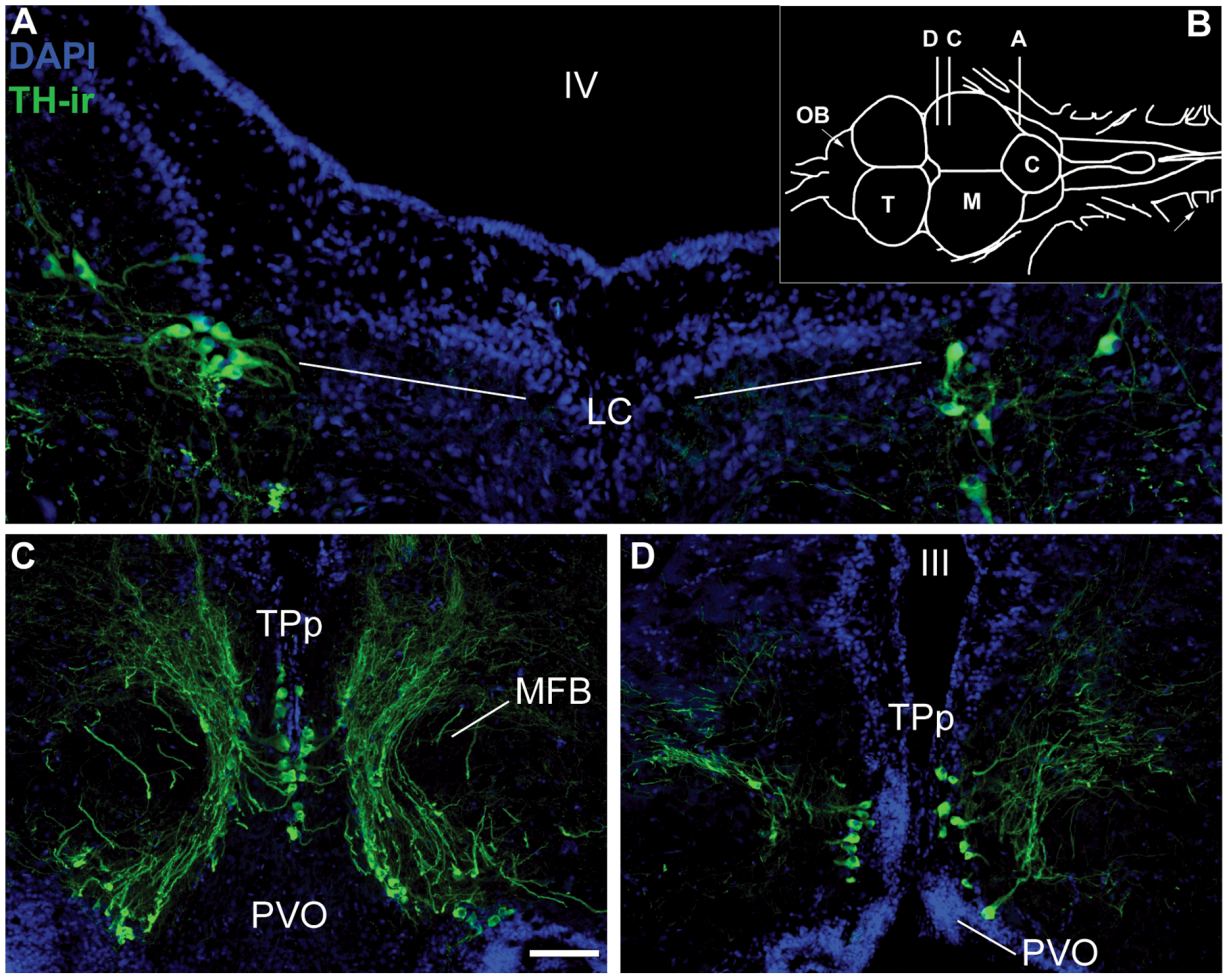
Catecholaminergic anatomy. Tyrosine hydroxylase (TH) immunoreactivity (ir) was used as a marker for catecholaminergic neural populations. (**A**) The bilateral noradrenergic locus coeruleus (LC). (**B**) (inset) Dorsal view of midshipman brain with relative positions of A, C, D. (**C**) Representative caudal (**C**) and rostral (**D**) sections of the dopaminergic periventricular posterior tuberculum (TPp). **Abbreviations:** Fourth ventricle (IV); Paraventricular organ (PVO); Third ventricle (III). Scale bar = 50 µm (LC) and 100 µm (TPp).

Activation of catecholaminergic neurons was measured by the occurrence of a cFos-ir nucleus within a TH-ir cell, which we refer to as colocalization [34]. To analyze the percentage of TH-ir cells colocalized with cFos-ir, photomicrographs were taken under the same conditions as those used for analysis in the auditory/vocal-acoustic centers. However, photomicrographs were taken with an additional filter set (FITC/CY2, Chroma, Bellow Falls, VT) within a z-stack of 9 levels (4 above and 4 below a central plane) each 1 µM thick to capture all TH-ir somata. Photomicrographs were combined into a single projected image in MetaMorph. The DAPI and green channels were overlaid, and TH-ir cells were counted manually. cFos-ir neurons were quantified using MetaMorph IMA in an identical manner to the auditory/vocal-acoustic centers. Using MetaMorph’s create region function, outlines were created around cFos-ir cells and transferred to the TH/DAPI overlaid image. We manually counted each instance where an outline indicating cFos-ir was located within the nucleus of a TH-ir cell, confirmed by DAPI nuclear stain. The sum of TH-ir cells that contained cFos-ir was divided by the total number of TH-ir cells for a percentage of TH/cFos-ir colocalization. Although TH is the rate-limiting enzyme in the production of all CAs, biochemical and genetic markers have substantiated TH-ir neurons in the LC and TPp as noradrenergic and dopaminergic, respectively, in fishes, consistent with their proposed homologies in other vertebrates [25], [27], [31]–[33], [39], [51]. The noradrenergic locus coeruleus (LC; Fig. 2A) was located by the presence of TH-ir cells dorsolateral to the medial longitudinal fasciculus (MLF) in the isthmal region between the hindbrain and midbrain [52]. Sampling began with the bilateral appearance of TH-ir cells and continued serially in the caudal to rostral direction for an average of 7.42 (±1.3 SD) sections per animal until their disappearance. A single photomicrograph was taken with a 20x objective of TH-ir neurons in each hemisphere. An independent samples t-test showed no difference in the numbers of sections analyzed between groups (*p*>0.2).

Analysis of cFos-ir colocalization within TH-ir neurons of TPp began caudally with the appearance of dense clusters of large, pear-shaped TH-ir cells medial to the medial forebrain bundle (MFB) that extended ventrolaterally along the lateral border of the paraventricular organ (PVO; Fig. 2C,D) [33], [53]. Up to three 20x photomicrographs were needed to capture all TH-ir cells per section and photomicrographs were aligned prior to analysis to avoid overlap. TPp was analyzed serially for an average of 7.4 (±1.9 SD) sections in the caudal to rostral direction until the disappearance of large, pear-shaped TH-ir cells adjacent to the midline. The caudal three sections of TPp accounted for the majority of TH-ir cells in the nucleus (paired-samples t-test, *p*<0.001, data not shown). One animal in the social signal group was excluded from analysis due to tissue damage that rendered the caudal portion of TPp unusable. The number of sections analyzed throughout the TPp did not differ between groups (independent samples t-test, *p*>0.3).

#### Statistics

Data for number of cFos-ir neurons per section and percent TH-ir neurons with cFos-ir nuclei were analyzed via independent samples t tests with males who were played back advertisement calls (social signal) and males who were not (ambient noise control) as between group factors. Levene’s test showed homogeneity of variance between groups for each independent variable analyzed (*p*>0.07 in all cases). We performed pair-wise correlations to investigate the relationships between the cFos response within auditory/vocal-acoustic nuclei and cFos colocalization within TH-ir cells of the LC, and TPp. Experimental groups were not added as a factor in Pearson correlations as all animals were exposed to an acoustic environment ranging from ambient environmental noise to advertisement call playback. All statistics were performed on IBM SPSS Statistics Version 19. We used the Benjamini-Hochberg correction [54] on our alpha level of *p = *0.05 for each t-test and pair-wise comparison performed, which we pooled to correct for multiple comparisons within the same data set. All statistics reported are significant relative to their corrected alpha level.

## Results

Morphological analyses were performed on all fish used in the study. For the social signal exposed males, standard length (SL) = 16.4±0.8 cm (mean ± SD), body mass (BM) = 74.5±10.2 g, and gonadosomatic index (GSI) = 0.82±0.31. For the ambient noise exposed males, SL = 17.0±1.3 cm, BM = 72.7±15.7 g, and GSI of 0.83±0.34. There were no differences in the body metrics (SL, BM, and GSI) between social signal and ambient noise exposed males (*p*>0.4 for all cases).

### Brain Activation of Ascending Auditory Nuclei

**Figure 3:**
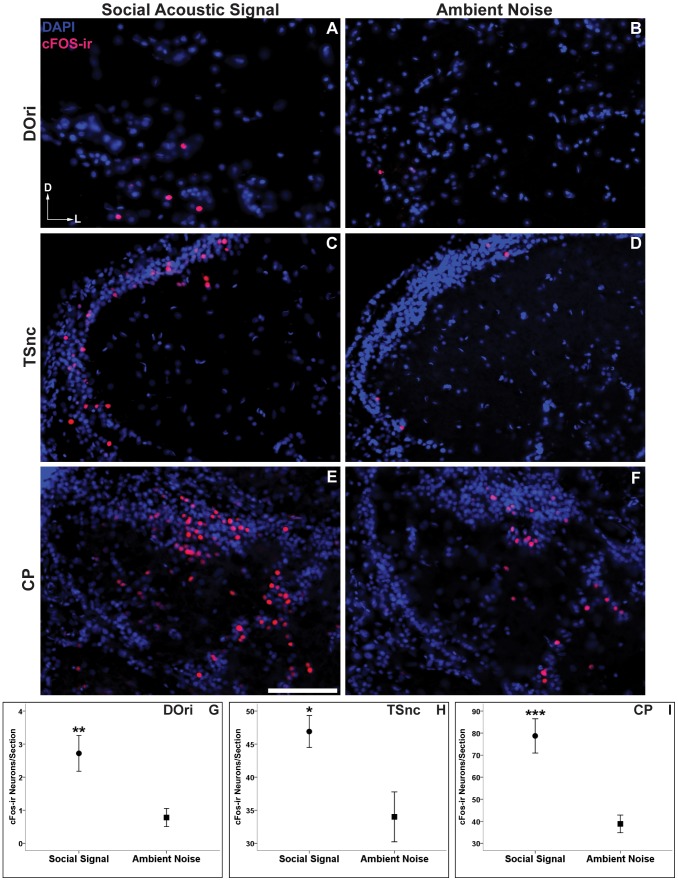
cFos response to social acoustic signals (advertisement calls) in the ascending auditory pathway. Representative images of cFos-ir neurons (red/pink) within males who were exposed to social acoustic signals (left column, A, C, E) versus males who were exposed to ambient environmental noise (right column, B, D, F). (**A**,**B**) Rostral intermediate division of the descending octaval nucleus (DOri) within the medulla. (**C**,**D**) The periventricular nucleus centralis within the midbrain torus semicircularis (TSnc). (**E**,**F**) Compact division of the central posterior nucleus (CP) in the auditory thalamus. Scale bar = 100 µm. Arrows represent the dorsal (D) and lateral (L) orientation for each image. Data in **G**–**I** are represented as mean number of cFos-ir neurons per section ± SE, *p<0.05 **p≤0.01, ***p<0.001.

Numbers of cFos-ir neurons were analyzed between groups in the hindbrain DOri (Fig. 3A–B), the midbrain TSnc (Fig. 3C–D), and 2 subdivisions of CP (CPc and CPd) (CPc shown in Fig. 3E–F) within the auditory thalamus. Males exposed to social acoustic signals had a significantly greater number of cFos-ir neurons in DOri (t(10) = 3.19, *p* = 0.01, Fig. 3G) and TSnc (t(10) = 2.88, *p*<0.05, Fig. 3H) than males exposed to ambient environmental noise. We found that social signal males also had significantly greater numbers of cFos-ir neurons in CPc (t(10) = 4.14, *p*<0.01), as well as CPd (t(10) = 4.68, *p* = 0.001). Values for both divisions of CP were combined in each section throughout the nucleus as a total measure of neural activity in the auditory thalamus. Social signal males were found to have more cFos-ir neurons in the auditory thalamus than ambient noise controls (CP (t(10) = 4.56, *p* = 0.001, fig. 3I). Taken together, these data show that males exposed to other male’s advertisement calls show a significantly greater activation at three levels of the ascending auditory system over exposure to ambient environmental noise.

### Brain Activation of Hypothalamic Vocal-Acoustic Nuclei

**Figure 4:**
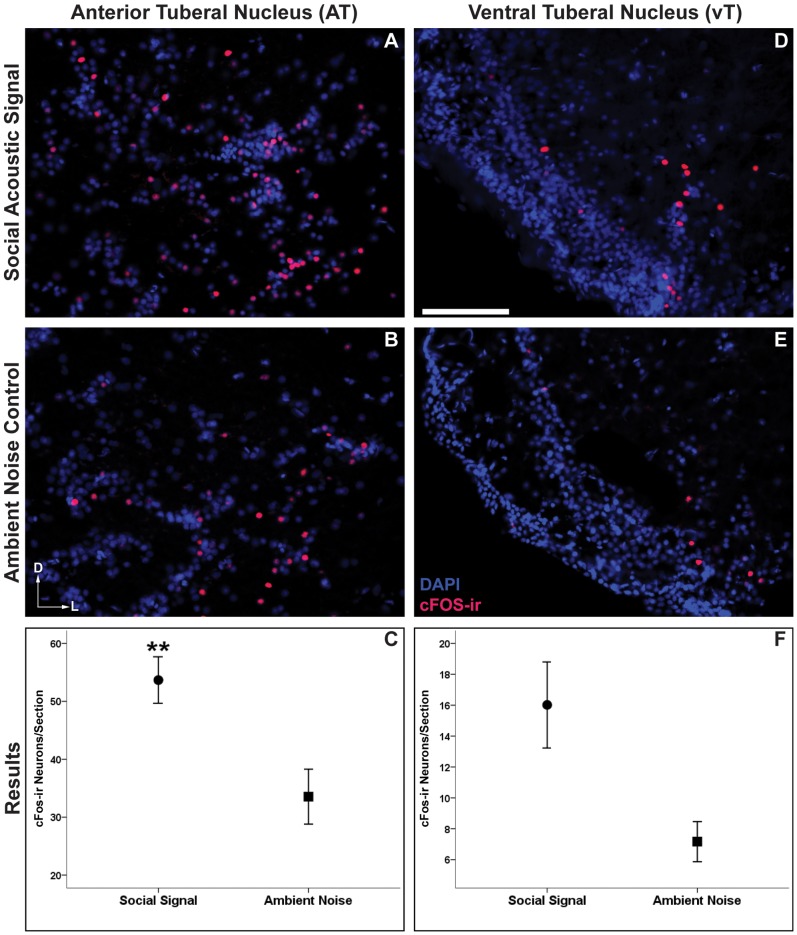
cFos response to social acoustic signals (advertisement calls) in hypothalamic vocal-acoustic circuitry. Representative images of cFos-ir neurons (red/pink) within the anterior tuberal nucleus (AT) (**A**,**B**) and ventral tuberal nucleus (vT) (**D**,**E**) of males who were exposed to social acoustic signals versus males exposed to ambient environmental noise. Data in **C** and **F** are represented as mean number of cFos-ir neurons per section ± SE, **p = 0.01. Scale bar = 100 µm. Arrows represent the dorsal (D) and lateral (L) orientation for each image.

Exposure to social acoustic signals had a significant effect on the numbers of cFos-ir neurons in one of the two SBN/vocal-acoustic nuclei that we analyzed. The hypothalamic AT (Fig. 4A–B) which connects to both the ascending auditory and descending vocal motor pathways showed a greater number of cFos-ir neurons in social signal over control males (t(8) = 3.24, *p* = 0.01, Fig. 4C). While the average number of cFos-ir neurons was higher in the vT of social signal males (Fig. 4D–E), this difference did not reach statistical significance (t(7) = 2.13, *p* = 0.07, Fig. 4F).

### Activation of CA Neurons

**Figure 5:**
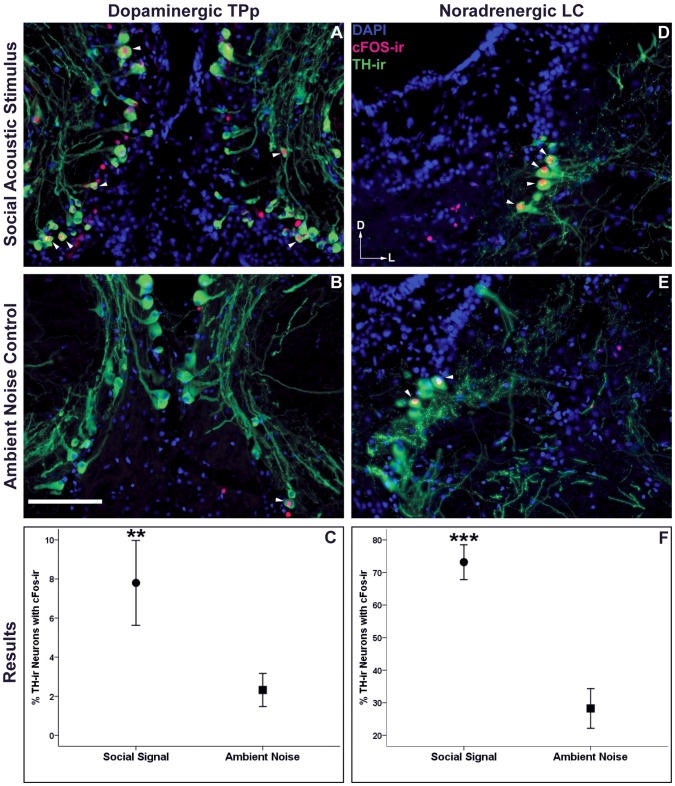
cFos-ir colocalization with catecholaminergic (TH-ir) neurons. Arrowheads indicate cFos-ir colocalized to catecholaminergic neurons within the dopaminergic periventricular posterior tuberculum (TPp) (**A, B**) and the noradrenergic locus coeruleus (LC) (**D,E**) of males exposed to social acoustic signals and males exposed to ambient noise. Data in **C** and **F** are represented as mean percent colocalization ± SE, **p≤0.01, ***p<0.001. Scale bar = 100 µm. Arrows represent the dorsal (D) and lateral (L) orientation for each image.

Catecholaminergic neurons within the NAergic LC (Fig. 5D–E) and the DAergic TPp (Fig. 5A–B) were differentially activated (as assayed by TH/cFos-ir colocalization) in male midshipman exposed to social acoustic signals over those exposed to ambient environmental noise. Males exposed to other male advertisement calls had a significantly greater percent colocalization of cFos-ir in TH-ir LC (t(10) = 5.53 *p*<0.001, Fig. 5F) and TPp neurons (t(9) = 3.21, *p* = 0.01, Fig. 5C) compared to controls. Importantly, there was no difference in the total number TH-ir neurons analyzed between groups in either the LC (Social Stimulus: 36.7 Ave TH-ir neurons ±6.9 SD, Ambient Noise: 43.0±10.1; *p*>0.2) or the TPp (Social Stimulus: 189±82, Ambient Noise: 211±51; *p*>0.5).

### Co-activation of Auditory/Vocal-Acoustic Nuclei and CA Neurons

**Figure 6:**
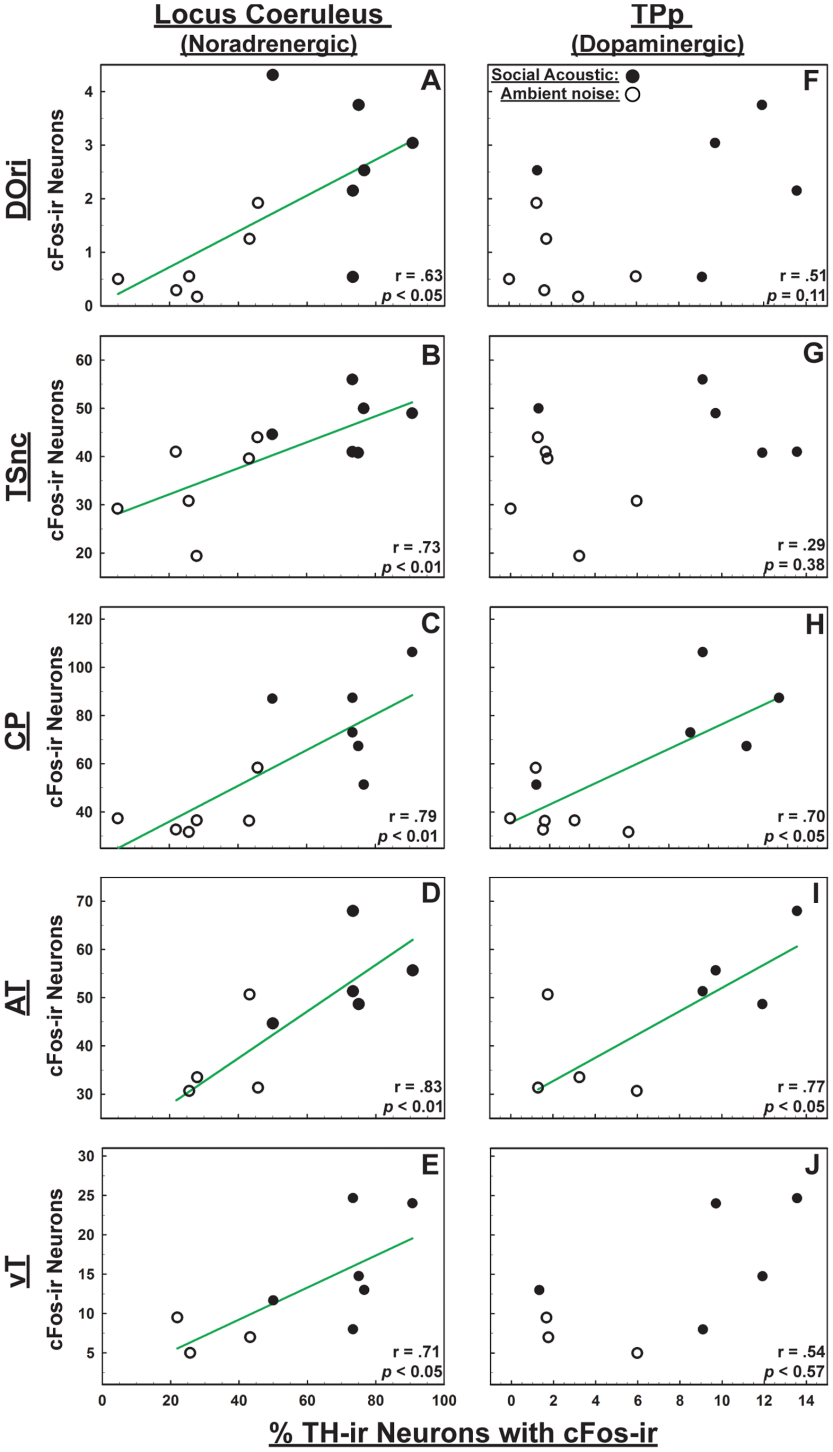
Co-activation of auditory/vocal-acoustic nuclei and CA neurons. Pairwise correlations between numbers of cFos-ir neurons in auditory/vocal acoustic nuclei and percent colocalization of cFos-ir within tyrosine hydroxylase (TH-ir) neurons of locus coeruleus (LC) (**A–E**) and the periventricular posterior tuberculum (TPp) (**F–J**). Closed circles are males exposed to social signals; open circles are males exposed to ambient noise; green trend lines indicate significant correlations (*p*≤0.05).

Finally, we performed pair-wise correlations to determine if there was a functional relationship between the number of cFos-ir neurons within the auditory/vocal acoustic circuitry with the percentage of TH-ir neurons colocalized with cFos-ir in the LC and TPp. In the ascending auditory pathway, LC had a significant positive correlation with DOri (r = 0.63, *p*<0.05 Fig, 6A), TSnc (r = 0.73, *p*<0.01 Fig. 6C) and CP (r = 0.79, *p*<0.01 Fig. 6E). TPp had significant positive correlation with CP (r = 0.70, *p*<0.05 Fig. 6F). There was no correlation between percent colocalization in TPp with DOri (r = 0.51 *p* = 0.11 Fig. 6B), or with TSnc (r = 0.29, *p* = 0.38 Fig. 6D). In the vocal-acoustic pathway, LC had a significant positive correlation with both AT (r = 0.83, *p*<0.01 Fig. 6G) and vT (r = 0.71, *p*<0.05 Fig. 6I), while TPp correlated positively only with AT (r = 0.77, *p*<0.05; vT: r = 0.54, *p* = 0.17 Fig. 6H/J respectively).

## Discussion

The localization of immediate early gene transcripts and products has been established as a reliable method for mapping the neural response to a variety of stimuli [55], [56] including auditory stimuli in mammals [57], birds [58]–[60] and anuran frogs [61]–[64]. Here we demonstrate that immediate-early gene products such as cFos can be used to map the neural response to social acoustic signals in teleost fish, the vertebrate group where vocal- acoustic communication is thought to have first evolved [10]. Our results corroborate numerous neurophysiological and neuroanatomical tract-tracing studies that have delineated the auditory [19], [21], [22],vocal-acoustic [9], [18], [38], [65] and social behavior network [6] circuitry of this species. We show that males exposed to social acoustic signals (advertisement calls of other males) had a greater number of cFos-ir neurons across several levels of the ascending auditory pathway, at least one level of the descending vocal-motor circuit, and in two key catecholaminergic neuronal populations compared to fish exposed to ambient noise. Importantly, these results are the first to show activation within catecholaminergic nuclei after exposure to a social acoustic stimulus in any fish, and, consistent with studies in tetrapods, support the hypothesis that catecholamines are important neuromodulators of vocal-acoustic communication across vertebrates.

### cFos-ir Response in Ascending Auditory Circuitry

As predicted, the social acoustic signal induced significant increases in numbers of cFos-ir cells in auditory processing centers in the hindbrain (DOri), midbrain (TSnc) and thalamus (CP) compared to ambient noise alone. The descending octaval nucleus (DO) is directly innervated by nVIII, projects to midbrain TS and represents a first-order auditory nucleus within the CNS of the midshipman fish [18], [19], as well as other teleosts [19], [66]–[69]. Importantly, in midshipman, adaptive reproductive-related changes in frequency encoding occur in the inner ear, implying that social incentive processes begin even at the level of the auditory periphery [13], [16], [17]. The rostral intermediate subdivision (DOri) that we analyzed in this study also has a direct connection to the prepacemaker nucleus of the vocal pattern generator and thus is designated as part of a hindbrain vocal-acoustic complex that can serve to provide feedback during calling [9], [18], [19], [70].

In anamniote vertebrates (e.g., cartilaginous and bony fishes and anuran frogs), tertiary auditory processing occurs in the torus semicircularis (TS) which is the midbrain homologue to the mammalian inferior colliculus [19], [61], [71], [72]. In the plainfin midshipman fish, the periventricular nucleus centralis (nc) has been physiologically identified as the primary auditory center within TS [19], and participates in the ability to discriminate between the physical properties of overlapping advertisement calls [21], [22]. Similar to our findings in midshipman, transcripts for *cfos* as well as another immediate early gene *egr-1* were found to be differentially increased in response to socially relevant auditory stimuli in various subdivisions of TS in male Túngara frogs (*Physalaemus pustulosus*) [61], [63]. Unlike the anuran TS which has multiple cytoarchitecturally distinct subdivisions with differing efferent and afferent connections within the auditory circuit [61], [71], [72], nc is the primary auditory center within TS of the midshipman [19]. The teleost TS does have functionally discrete subregions such as the nucleus ventrolateralis (TSnv) which is the midbrain target of the ascending lateral line system [73]. TSnv is both anatomically and functionally distinct from TSnc, and was excluded from our analysis.

TSnc supplies the primary auditory input into the compact and diffuse divisions of the central posterior nucleus (CPc and CPd) within the thalamus [9], [19]. Whether or not CPc and CPd serve discrete roles in auditory processing and integration is unknown. To date, only Lu and Fay [74] have recorded from teleost neurons in the CP and their results suggest a function of CP different from TS, with perhaps the auditory thalamus involved more in the integration of frequency-selective channels for processing more complex, wideband spectral features of natural or socially-relevant sound sources. Increases in e*gr-1* transcripts within the auditory thalamus of the Túngara frog have been shown to relate to a behavioral (locomotor) response to auditory stimuli which suggests the thalamus as a site for sensorimotor integration [75]. We found a greater difference in cFos response in the CP of social signal recipients vs. ambient noise compared to differences in lower auditory centers, suggesting possible higher-order auditory functions, e.g. [74]. The role of CP as a sensorimotor integration center in response to conspecific auditory signals in teleost fishes warrants further study [75].

### cFos-ir Response in Vocal-Acoustic Circuitry/Nodes of SBN

Both the anterior (AT) and ventral (vT) tuberal hypothalamus comprise part of the midshipman descending vocal-motor system where vT is the most robust forebrain vocal stimulation site [76]–[78]. vT receives input from CP, however it is not considered part of the acoustic circuitry. AT shares reciprocal connections with CP and receives strong projections from TS and is therefore also considered part of the ascending auditory system [9], [19], [67]. The differentially stronger input from the auditory system may explain, in part, the greater cFos response in AT versus vT to the social acoustic signal over ambient noise.

In order to assess the social relevance of a stimulus, integration of environmental cues and internal state must occur within the CNS [9], [79], [80] and the social behavior network (SBN) represents a suite of nuclei which may coordinate the production of an appropriate behavioral response to such cues [5]–[7]. The teleost AT and vT are thought to be partially homologous to the mammalian and avian ventromedial hypothalamus (VMH) and anterior hypothalamus (AH), respectively [6], [8], [81] which are designated nodes in the SBN. Recently, Maruska and colleagues [80] showed that levels of IEG transcripts (both *cfos* and *egr-1*) were elevated in AT and vT of African cichlid (*Astatotilapia burtoni*) males during social ascension from a subordinate to a dominant phenotype. In that study social ascension was triggered by the removal of an established dominant male from the environment, and the increase in IEG transcripts in AT and vT (as well as every other node of the SBN) was attributed to the perception of a social opportunity [80]. As midshipman are nocturnal, we tested type I males at night when they typically court females [11] and we manipulated only their acoustic environment (playback of advertisement calls vs. ambient noise) during our experiments. Hearing another male’s advertisement call may be a signal that causes activation of AT within the neural circuit(s) responsive to a social opportunity in the context of courtship and reproduction.

While type I males build and defend nests, their mate call is not typically displayed during direct agonistic encounters [14]. Alternatively, hearing another male’s mate call within a competitive social context could be perceived as a challenge and cause a neural response pattern associated with territoriality or competition. In mammals, cFos responses within the VMH have implicated this nucleus in neural circuits dedicated to both agonistic and mating behavior [82]. In a territorial Estrildid finch, the violet-eared waxbill (*Uraeginthus granatinus*), increased cFos-ir (as well as EGR-1-ir) neurons are found in the VMH and AH after exposure to a male conspecific [83]. In breeding condition, male European starlings (*Sturnus vulgaris*) behave territorially when in possession of a nest box from which to vocally court females, and have a higher baseline level of cFos-ir neurons in VMH and AH than do males without nest boxes [84]. Interestingly, the number of cFos-ir neurons in VMH and AH had a significant positive relationship with total number of songs produced by breeding condition males regardless of whether or not they possessed a nest box [84]. The ability to perceive social acoustic stimuli is thought to be adaptive only if it facilities an appropriate behavioral response [8]. Previously, it was demonstrated that “challenging” male Gulf toadfish with a playback of other male advertisement calls changes their internal physiological state (increases levels of circulating 11-ketotestosterone and cortisol), and causes an increase in both the rate and duration of a vocal response [23]. Our results suggest AT may play an important role for integrating the natural social acoustic environment with a subsequent vocal response [9], [23]; however, we were unable to monitor vocalizations in response to mate-call playbacks in this experiment. Future studies will be needed to identify behavioral responses of males to social acoustic signals in this species.

### cFos-ir Response in TH-ir Neurons in the TPp and LC

While circulating steroid hormones are documented to increase during playback of advertisement calls in male toadfish (see above), they are unlikely to be the only chemical responders to such a social challenge. Levels of the catecholamines DA and NE have been shown to increase in the brains of the lizard (*Anolis carolinensis)* during presentation of an actual [85] or simulated [86] opponent. Similarly, male Lincoln’s sparrows exposed to challenging songs of other males showed increased noradrenaline metabolites in the auditory forebrain and may be involved in the modulation of a motivated behavioral response to such a challenge [87]. DA has been implicated in motivated sexual behavior [36], [88] including appetitive sexual behavior in the form of female directed vocalizations in zebra finches [35], [89], [90] and European starlings (*Sturnus vulgaris)* [91]. Additionally, much attention has been paid to DA’s involvement in how the female brain responds to conspecific male vocalizations [48], [92]–[94]. Pharmacological depletion of DA neurons within the posterior tuberculum of female grey treefrogs (*Hyla versicolor*) decreased phonotaxis to male advertisement calls, implicating this nucleus in auditory and/or motor behavior in the anamniote brain [95]. However, to our knowledge the present study is the first to demonstrate that specific groups of dopaminergic and noradrenergic neurons are responsive to merely “hearing” acoustic signals from one’s potential competitor.

The large, pear-shaped TH-ir neurons within the diencephalic TPp have been substantiated biochemically and genetically as dopaminergic [51], [53], and are the major source of far-reaching (i.e., non-local) DA projections ascending into the ventral telencephalon (including striatal homologues), and descending into the hindbrain and spinal cord of teleost fish [31], [33], [39], [51], [96]. Teleosts lack an ascending DA system that originates in the midbrain as found in cartilaginous fishes and tetrapods [27], [32], [97]. While there is strong anatomical and genetic evidence that TPp is homologous to the A11 dopaminergic group found in amniotic vertebrates (see [32] and refs within), hodological evidence has lead to the proposal that TPp may serve a functionally analogous role, at least in part, to the amniote ventral tegmental area (VTA), a key nucleus in the mesolimbic reward system [8], [33]. Importantly, because TPp’s chemical and hodological characteristics may allow it to modulate behavior by serving as a sensorimotor integration center, it is a good candidate nucleus within the teleost brain to test the hypothesis that DA is involved in the neurological response to a playback challenge and auditory-driven social behavior. Our hypothesis that DAergic neurons would be involved in the neural response to a playback challenge was supported by a significant increase in the percentage of TH-ir cells that were colocalized with cFos-ir in the TPp of males exposed to other males’ advertisement calls. Our results show DA’s involvement in the neural response to social acoustic signals in a teleost fish and these data are consistent with the response of DAergic neurons within both the diencephalon and midbrain to social and vocal-acoustic stimuli in other vertebrates [34], [37], [90].

A significant positive correlation between percent cFos induction in TPp TH-ir neurons and in the number of cFos-ir neurons in CP and AT supports possible functional connectivity of these nuclei [62]. TPp, however, does not appear to receive direct efferent projections from the auditory system [19], but rather is indirectly connected to the auditory system via reciprocal connections with the periaqueductal gray (PAG), a midbrain vocal-acoustic center and node of the SBN [6], [9], [38], [65]. Additionally, multiple nuclei within SBN as well as auditory and vocal-motor circuits (including TS, CP, AT, and vT) project to PAG [38]. These nuclei may produce specific patterns of combined input into PAG [5], [98] that may be needed to gait activation of TPp in auditory-driven social behavior. As some DAergic cells within TPp send simultaneous ascending and descending projections [31], [99], TPp may add salience to acoustic stimuli by both modulating sensory perception and coupling it with higher-order decision making and/or motivational processes [8].

Cues from one’s social-acoustic environment can lead to detection of conspecific competitors [23], [100] or potential mates [13], [15], [94]. In midshipman fish, seasonal auditory plasticity in females is steroid-dependent and improves peripheral auditory encoding to better detect the male advertisement call [17]; however, the neurochemicals that potentially modulate the CNS in both females and males to mediate behavioral responses to social signals are less understood [101]. The catecholamine NE has been implicated in general arousal, selective attention, and neural encoding of salient sensory stimuli [26], [102]. The locus coeruleus (LC) within the isthmus of the rostral hindbrain represents the primary ascending NEergic system in teleost fish [31], [32], [52], [103], and its projection targets are highly conserved throughout vertebrates and include the auditory midbrain [27], [31].

We report a greater percentage of TH-ir cells colocalized with cFos-ir within LC of males exposed to other male advertisement calls, which is consistent with results found in female zebra finches exposed to male courtship vocalizations [104]. We also report that cFos induction in the LC had a significant positive correlation with number of cFos-ir neurons at all levels of the auditory and vocal-acoustic pathways that we analyzed. A positive correlation with percent colocalization in LC with cFos-ir neurons in DOri as well as TSnc, CP, AT, and vT, is consistent with NE’s involvement in neural arousal [29]. A positive correlation between neural activity within LC and acoustic circuits is consistent with the hypothesis that NE is needed for both auditory processing and the discrimination of social acoustic signals [87]. Pharmacological depletion of NE projections with N-(2-chloroethyl)-N-2-bromobenzyl-amine hydrochloride (DSP-4) has been shown to reduce the IEG (ZENK) response in multiple forebrain nuclei of female zebra finches [105], as well as the behavioral response to sexually stimulating songs when they were played back to female canaries with increasing levels of white noise [106]. The data in the present study similarly demonstrate a neural response in LC to social acoustic signals in a teleost. Future studies will be needed to elucidate the roles that both noradrenaline and dopamine play in social acoustic and sociosexual behavior in teleost fishes. Overall our data support a conserved and integral role for catecholamines in vocal-acoustic social behavior across vertebrate taxa.
